# Skeletal Muscle Is an Early Site of Zika Virus Replication and Injury, Which Impairs Myogenesis

**DOI:** 10.1128/JVI.00904-21

**Published:** 2021-10-27

**Authors:** Daniel Gavino-Leopoldino, Camila Menezes Figueiredo, Mariana Oliveira Lopes da Silva, Letícia Gonçalves Barcellos, Rômulo Leão Silva Neris, Laryssa Daniele Miranda Pinto, Suzana Maria Bernardino Araújo, Leandro Ladislau, Claudia Farias Benjamim, Andrea T. Da Poian, Julia Rosauro Clarke, Claudia Pinto Figueiredo, Iranaia Assunção-Miranda

**Affiliations:** a Instituto de Microbiologia Paulo de Goes, Universidade Federal do Rio de Janeiro, Rio de Janeiro, Brazil; b Faculdade de Farmácia, Universidade Federal do Rio de Janeiro, Rio de Janeiro, Brazil; c Instituto de Ciências Biomédicas, Universidade Federal do Rio de Janeiro, Rio de Janeiro, Brazil; d Instituto de Biofísica Carlos Chagas Filho, Universidade Federal do Rio de Janeiro, Rio de Janeiro, Brazil; e Instituto de Bioquímica Médica Leopoldo de Meis, Universidade Federal do Rio de Janeiro, Rio de Janeiro, Brazil; University of North Carolina at Chapel Hill

**Keywords:** Zika virus replication, skeletal muscle, viral dissemination, muscle inflammation, myogenesis, pathogenesis

## Abstract

Zika virus (ZIKV) infection became a worldwide concern due to its correlation with the development of microcephaly and other neurological disorders. ZIKV neurotropism is well characterized, but the role of peripheral viral amplification to brain infection remains unknown. Here, we found that ZIKV replicates in human primary skeletal muscle myoblasts, impairing its differentiation into myotubes but not interfering with the integrity of the already-formed muscle fibers. Using mouse models, we showed ZIKV tropism to muscle tissue either during embryogenesis after maternal transmission or when infection occurred after birth. Interestingly, ZIKV replication in the mouse skeletal muscle started immediately after ZIKV inoculation, preceding viral RNA detection in the brain and causing no disruption to the integrity of the blood brain barrier, and remained active for more than 2 weeks, whereas replication in the spleen and liver were not sustained over time. In addition, ZIKV infection of the skeletal muscle induces necrotic lesions, inflammation, and fiber atrophy. We also found a reduction in the expression of regulatory myogenic factors that are essential for muscle repair after injury. Taken together, our results indicate that the skeletal muscle is an early site of viral amplification and lesion that may result in late consequences in muscle development after ZIKV infection.

**IMPORTANCE** Zika Virus (ZIKV) neurotropism and its deleterious effects on central nervous system have been well characterized. However, investigations of the initial replication sites for the establishment of infection and viral spread to neural tissues remain underexplored. A complete description of the range of ZIKV-induced lesions and others factors that can influence the severity of the disease is necessary to prevent ZIKV’s deleterious effects. ZIKV has been shown to access the central nervous system without significantly affecting blood-brain barrier permeability. Here, we demonstrated that skeletal muscle is an earlier site of ZIKV replication, contributing to the increase of peripheral ZIKV load. ZIKV replication in muscle promotes necrotic lesions and inflammation and also impairs myogenesis. Overall, our findings showed that skeletal muscle is involved in pathogenesis and opens new fields in the investigation of the long-term consequences of early infection.

## INTRODUCTION

Zika virus (ZIKV) is an arbovirus of the *Flaviviridae* family that is transmitted mainly by *Aedes* mosquitoes. ZIKV has disseminated rapidly across the Americas in recent years (1) and, although historically this infection caused a self-limited mild febrile condition similar to that induced by other arboviruses, reports of persistent and severe neurological damage caused by ZIKV infection have emerged more recently. It is now known that vertical transmission of ZIKV can cause fetal death and congenital defects, such as microcephaly and other neurological complications (2), while infection in adults has been associated with the development of neuroinflammatory disease, such as Guillain-Barré syndrome, acute myelitis, and encephalitis (3, 4).

Several studies on the mechanisms of ZIKV infection pathogenesis focused on the damage caused by virus replication in the central nervous system (CNS). It has been demonstrated that ZIKV replicates in human neural progenitor cells and brain organoids, resulting in cell death and cell cycle arrest (5). In mice, both intrauterine and neonatal exposure to ZIKV impaired neurogenesis and caused severe necrosis in different brain regions, along with persistent viral replication and neuroinflammation (6, 7). Moreover, it has been shown that virus-induced activation of microglial cells and astrocytes affected cognitive function and the differentiation of glial progenitor cells, impairing brain development (8, 9).

Many neurotropic pathogens reach the CNS because infection interferes with the integrity of the highly selective endothelial layer that forms the blood-brain barrier (BBB). In contrast, ZIKV has been shown to cross the BBB without significantly affecting its permeability (10, 11). In addition, it was demonstrated that ZIKV replicates in human peripheral neurons *in vitro* (12). Thus, an interesting hypothesis to be considered is that ZIKV replication in motor and sensory tissues would be a route used by ZIKV to access the peripheral nerves, reaching the brain by retrograde axonal transport. This is a mechanism known to be involved in CNS invasion by other neurotropic viruses, including the West Nile virus, which is also member of the *Flaviviridae* family (13–15). Despite the evidence that ZIKV can be detected during both acute and late phases of infection in several body fluids, eyes, testes, and vaginas (16), the importance of initial replication sites in viral spread to the CNS remains unknown.

Here, we investigated the ability of ZIKV to establish a productive replication in the skeletal muscle. We found that ZIKV replicates in human muscle precursor cells, impairing fiber differentiation. Using mouse models, we showed ZIKV tropism to muscle tissue either during embryogenesis or when infection occurred after birth. Interestingly, ZIKV replication in the muscle precedes the detection of viral RNA in the brain and induces necrotic lesions, inflammation, fiber atrophy and a reduction in the expression of myogenic factors. Taken together, our results indicate that the skeletal muscle is an early site of viral amplification and injury impairing myogenesis.

## RESULTS

### ZIKV replicates in human skeletal muscle cells.

**FIG 1:**
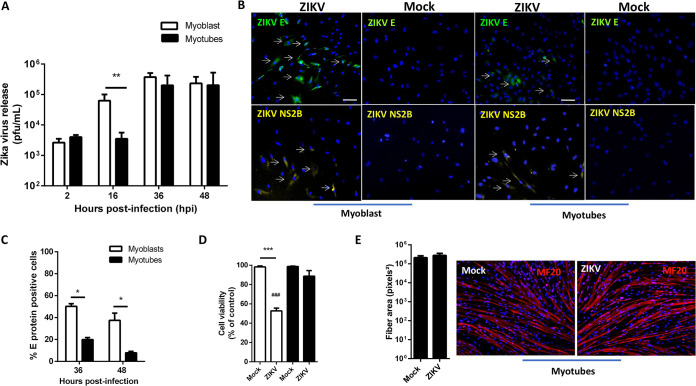
ZIKV replicates in human skeletal muscle cells. Human primary skeletal myoblast and differentiated myotube cultures were infected with ZIKV at an MOI of 5 and temporally assessed. (A) ZIKV released by myoblast (white bars) and myotubes (black bars) at different times postinfection was quantified in culture supernatants by using a plaque assay (*n* = 4 to 5 for each point). (B) Immunofluorescence analysis for the detection of ZIKV-positive myoblasts and myotubes at 36 hpi using anti-flavivirus E protein 4G2 and anti-NS2B of ZIKV. White arrows indicate some positive cells. (C) Quantitative analysis of 4G2-positive cells expressed as the percentage of the total (nuclear staining with DAPI) was performed using at least 10 fields of two independent experiments with ImageJ software. (D) Cell viability was determined by MTT reduction at 48 h after ZIKV infection relative to mock-treated cells for three independent experiments performed in triplicate. (E) Detection and quantification of fibers for mock- and ZIKV-infected differentiated myotube cultures were performed using staining with anti-myosin heavy chain (MF20) at 48 hpi. For quantification of the fiber area, the fluorescence for at least 10 fields of three independent experiments was determined and normalized by the total number of cells (nuclear staining with DAPI) using ImageJ software. Data were analyzed using two-way ANOVA (A and C) or one-way ANOVA (D), followed Sidak and Turkey multiple comparisons, respectively (*, *P* < 0.05; **, *P* < 0.01; ***, *P* < 0.001; ###, *P* < 0.001 [compared to ZIKV-infected groups]).

The peripheral sites of ZIKV replication and their role in neuronal infection are still poorly understood. To determine whether muscle tissue is a site of ZIKV infection, we first evaluated viral replication in primary culture of human skeletal muscle myoblasts (HSMMs), both before and after differentiation into myotubes (muscle fibers). Figure 1A shows that a productive ZIKV replication occurs in cultures of both myoblasts and myotubes. Release of infectious particles from myotubes occurred later than in myoblasts, but in both cells the viral titer in the culture medium increases more than 2 orders of magnitude by 36 h postinfection (hpi). In agreement with the replication curves, viral protein expression monitored by immunostaining against E or NS2B proteins was observed in both myoblasts and myotubes, whereas no staining was detected in mock-treated cultures (Fig. 1B). Quantification of E protein expression showed a significantly higher number of ZIKV-positive cells in myoblasts than in myotubes at 36 and 48 hpi (Fig. 1C). Interestingly, ZIKV replication greatly affected the viability of myoblasts but not myotubes in culture (Fig. 1D). Consistent with these observations, the fiber area was not altered after infection, as shown by comparing myosin heavy-chain (MF20) immunolabeling in mock- and ZIKV-treated myotube cultures (Fig. 1E), suggesting that ZIKV infection does not interfere with the integrity of terminally differentiated myofibers.

### ZIKV infection inhibits myogenesis.

**FIG 2:**
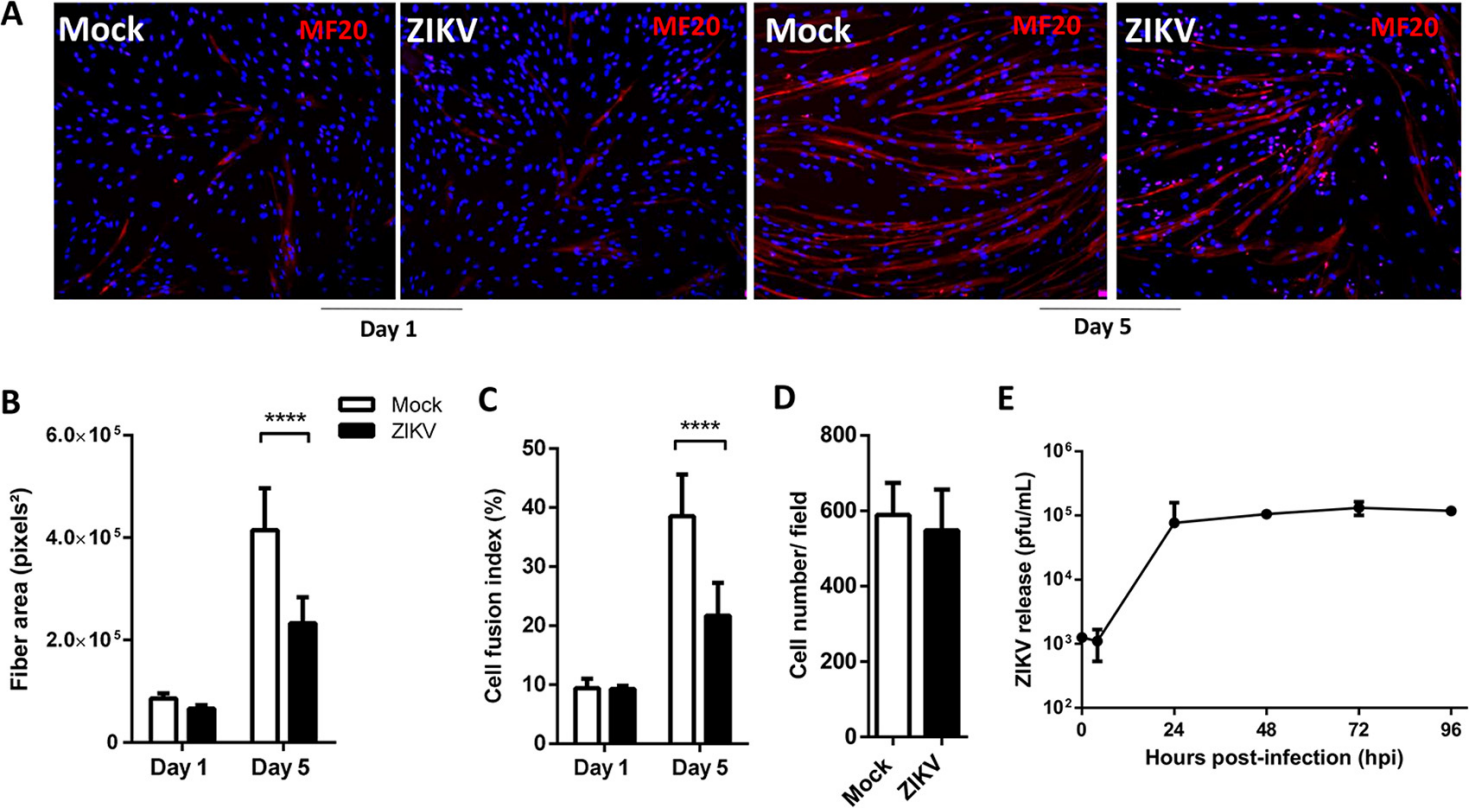
ZIKV infection inhibits myogenesis. Skeletal muscle progenitor cells were subjected to differentiation into myotubes. At day 1 of differentiation, cells were mock or ZIKV infected at an MOI of 1 and then cultured until day 5 of differentiation. (A) Cells were fixed at days 1 and 5 of differentiation, and formed fibers were detected by immunofluorescence using MF20 (red); the cell nucleus was stained with DAPI (blue). Fluorescence images were quantified using at least 10 fields of three independent experiments with ImageJ software to obtain the fiber area (B), the fusion index (%) (C), and the cell number per field (D) for mock (white bars)- and ZIKV (black bars)-infected cells. Positive nuclei were used for counting on each field and to normalize the values per field. (E) ZIKV released at culture supernatant at different times postinfection was quantified by plaque assay (*n* = 3, each point). Statistical analysis was performed by two-way ANOVA (****, *P* < 0.0001).

To investigate whether ZIKV replication interferes with myogenesis, we infected HSMM cells at an early stage of differentiation (1 day after incubation with differentiation-inducing medium) and evaluated fiber formation by performing MF20 immunolabeling shortly after infection (day 1) and when cultures reached the late stage of differentiation (day 5). ZIKV-infected culture showed a reduction of MF20-positive cells at day 5 compared to mock-treated cells (Fig. 2A). Quantitative analyses of MF20 staining indicated a significant decrease in the average fiber area (Fig. 2B) and in the number of differentiated fibers (Fig. 2C). The total number of cells per field did not change (Fig. 2D), suggesting that rather than causing the death of myotubes or arresting myoblast proliferation, ZIKV infection inhibits myoblast differentiation. We also observed a rapid increase in ZIKV replication in myoblasts under differentiation, with virus titers maintained for at least 96 hpi (Fig. 2E).

### ZIKV shows muscle tropism during embryogenesis.

**FIG 3:**
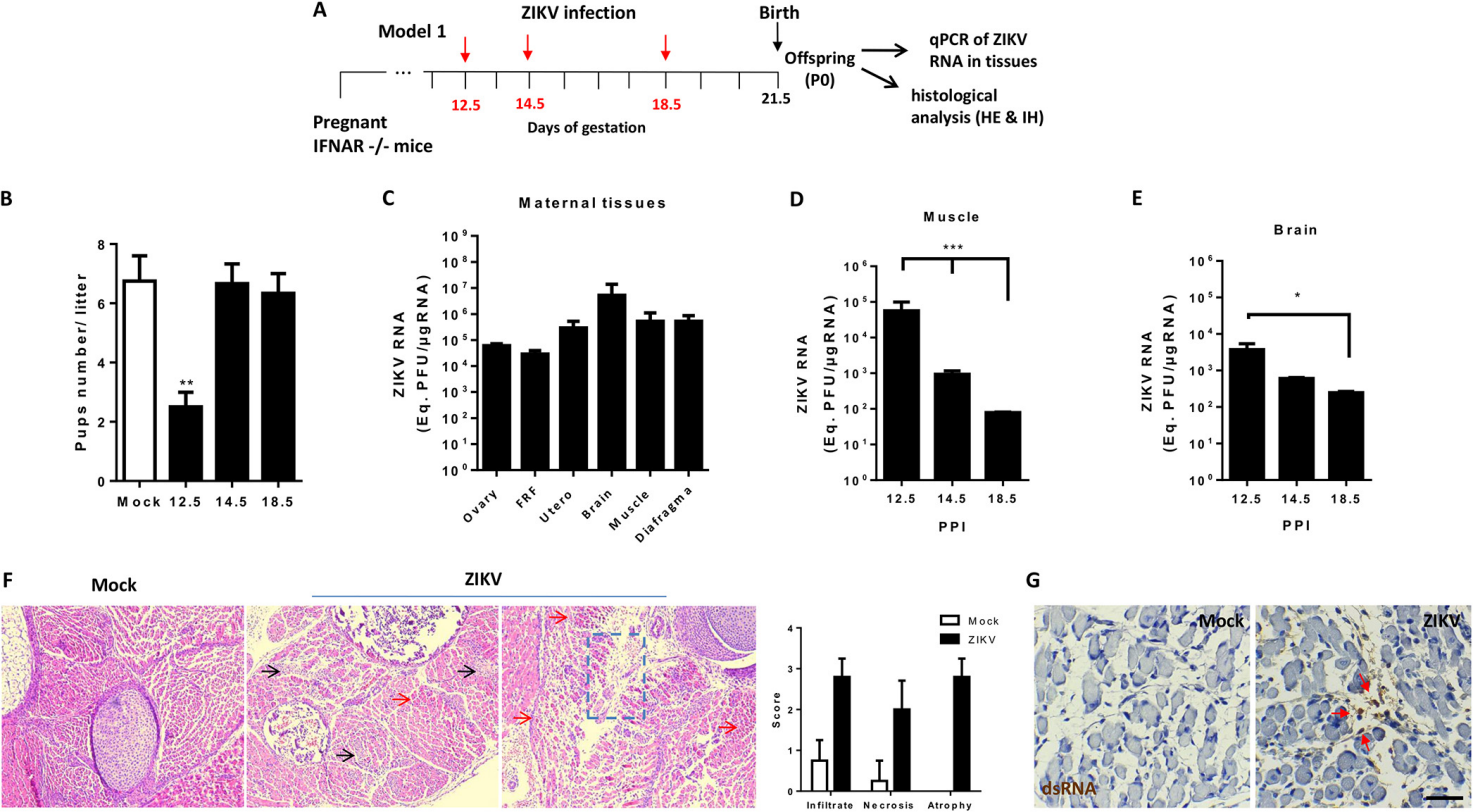
ZIKV shows muscle tropism during embryogenesis. (A) Schematic representation of the period of ZIKV infection with pregnant SVA129 mice (red arrow). For this model (model 1), SVA129 females were mock or ZIKV infected (10^5^ PFU) at different pregnancy periods (12.5, 14.5, and 18.5 days of gestation, at least *n* = 3 each), and tissues were analyzed at birth. (B) Numbers of pups per littermate after maternal exposure to mock or ZIKV infections at different pregnancy periods of infection (PPI). (C) Different tissues from female infected at day 12.5 of pregnancy were collected after delivery, and ZIKV RNA was quantified by qPCR (*n* = 4 to 6, each point). (D and E) Skeletal muscle from the hind legs (D) and brains (E) of pups were collected at birth after ZIKV inoculation at different PPI. ZIKV RNA in tissue was quantified by qPCR in samples from at least three independent experiments. (F) Muscle samples from pups either mock infected or at 12.5 PPI were fixed for histological analysis. The muscle tissues were embedded in paraffin after dehydration, and tissue sections of 5 μm were prepared, stained with H&E, and scored. Black arrows indicate areas of inflammation, red arrows indicate atrophy areas, and dashed lines indicate areas of lesion. (G) The ZIKV replication intermediary double-stranded RNA was stained in pup muscle sections at birth in mock- and ZIKV-infected groups with an anti-dsRNA J2 antibody. Slides were imaged on Sight DS-5M-L1 digital camera (Nikon, Melville, NY) connected to an Eclipse 50i light microscope (Nikon). Red arrows indicate positive staining areas. Values are plotted as means ± the standard errors of mean (SEM). Statistical analysis was performed by one-way ANOVA, followed of Tukey’s multiple-comparison test (*, *P* < 0.05; **, *P* < 0.01; ***, *P* < 0.001).

To investigate ZIKV tropism to the developing skeletal muscle during embryogenesis, we used a mouse model of ZIKV vertical transmission, in which type I interferon receptor-deficient (IFNAR^−/−^) pregnant mice were infected subcutaneously at different gestational periods (Fig. 3A). Infection at the early stages (until 12.5 days), but not at 14.5 of 18.5 days of gestation, strongly affected the gestation outcome, with a significant offspring reduction (Fig. 3B), probably due to fetal death, as shown by the presence of fetal resorption fragments (FRFs) together with amniotic sac and placental residues, as well as by cases of stillbirths (not shown). Shortly after delivery, high levels of ZIKV RNA were still detected in several maternal tissues, including ovaries and the uterus, and also in FRFs collected from dams infected at 12.5 days of gestation (Fig. 3C), supporting a deleterious effect of ZIKV on fetuses. To evaluate whether ZIKV reached the fetal muscle and replicated in this tissue during mouse development, skeletal muscles samples were collected bilaterally from the hind legs of pups on the day of birth and used for ZIKV RNA quantification by qPCR. We found that although viral titers were higher when infection was performed at earlier stages of pregnancy, ZIKV RNA was detected in pups muscle tissue also when infection was performed at later gestational times (Fig. 3D). ZIKV RNA was also detected in brains of newborn pups, with viral loads comparable to those found in muscle (Fig. 3E). Histological analysis of pup muscles revealed inflammatory infiltrates and necrotic areas, as well as muscle atrophy (Fig. 3F). In addition, reinforcing the evidences for ZIKV replication in the fetal muscle, we detected the replication intermediary double-stranded RNA (dsRNA) by immunohistochemistry (Fig. 3G), as well as ZIKV negative-strand RNA in the muscle of all pups after delivery (not shown). dsRNA detection was predominant in immature cells and in areas of reduced-caliber fibers and of lesion, as indicated by the arrows in the Fig. 3G. Taken together, these results indicate that after vertical transmission, ZIKV can reach and replicate in fetal skeletal muscle, maintaining high viral titers until the birth of the offspring.

### Muscle is a main site of peripheral ZIKV replication *in vivo*, preceding viral neuroinvasion.

To evaluate viral spread among different tissues during ZIKV infection in early life, we adapted a mouse model of neonatal subcutaneous infection previously described by our group (6). In order to maintain the same background of the maternal-transmission IFNAR^−/−^ model, we used wild-type (WT) SV129 mice instead of the Swiss mice used in our previous study. The ZIKV-induced disease in this mouse background was severe, leading to a reduction in body weight gain after 12 days of infection and 100% mortality 19 days after ZIKV inoculation (not shown).

**FIG 4:**
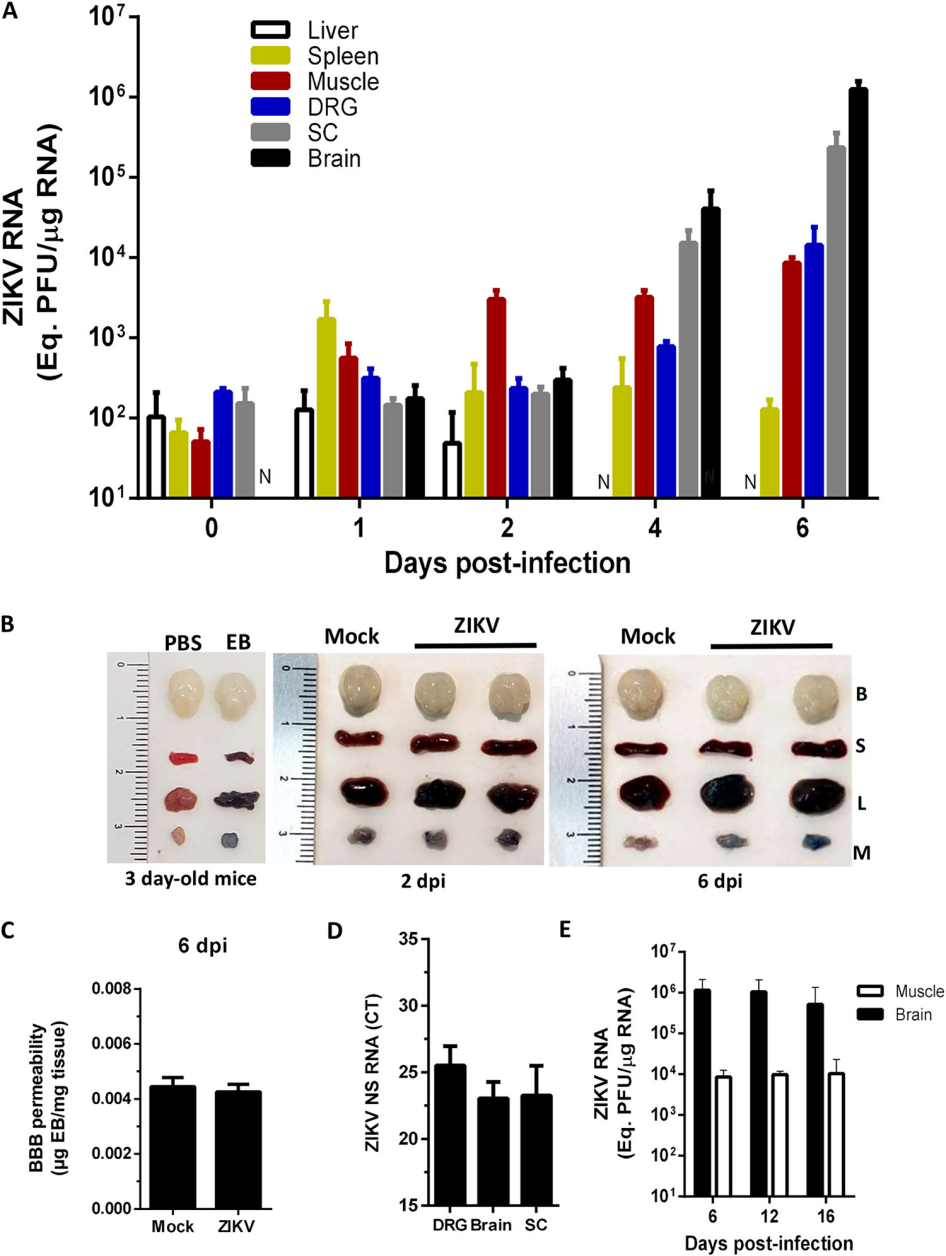
Muscle is a main site of peripheral ZIKV replication *in vivo*, preceding brain infection. (A) Three-day-old WT SV129 mice were ZIKV infected (10^6^ PFU), and the replication kinetics in skeletal muscle from the hind legs, livers, spleens, brains, spinal cords (SC), and dorsal root ganglia (DRG) were determined by qPCR (*n* = 4 to 6, each point). N, nondetectable. (B) Blood-brain barrier permeability was analyzed in WT mice before infection (3 day old) and in mock- or ZIKV-infected mice after 2 and 6 days. These mice were subcutaneously inoculated with 0.1% Evans Blue solution or PBS. After 1 h, the animals were perfused with PBS, and tissues were removed for visualization. EB, Evans Blue solution; B, brain; S, spleen; L, liver; M, muscle. (C) Evans’s Blue incorporated in brain tissue at 6 dpi was recovered in formamide solution, and the amount was determined by spectrophotometry. (D) Negative strand amplification was detected by qPCR in neural structures at 6 dpi, and the *C_T_* (cycle threshold) values were plotted. (E) The ZIKV load was detected at late times postinfection in muscle and brain by qPCR. Values are plotted as means ± the SEM.

Animals were infected on postnatal day 3, and the time course of viral replication was determined in skeletal muscle, spleen, and liver, as well as in the brain, dorsal root ganglion (DRG), and spinal cord (SC) (Fig. 4A). ZIKV RNA quantification in the skeletal muscle collected bilaterally from hind legs showed that viral amplification started immediately after virus inoculation, with a 10-fold increase in viral titer at 1 day postinfection (dpi) and the replication peak occurring at 2 dpi, plateauing for at least 6 dpi. We also observed an increase of ZIKV titers in spleen at 1 dpi, but in this case the titers decreased to a very low level in the following days. ZIKV titers in the liver did not increase over time and decreased to undetectable values by 4 dpi.

The rapid ZIKV replication in muscle tissue contrasted with the time course of viral dissemination in the nervous system. An increase in ZIKV RNA levels was detected only at day 4 of infection in the DRG, SC, and brain (Fig. 4A). These findings indicate that the muscle is a main site of peripheral ZIKV amplification and suggest a role for this tissue in viral neuroinvasion.

The absence of virus replication in the brain at early times after infection is consistent with the preservation of BBB integrity at the moment of ZIKV inoculation (3 days after birth; Fig. 4B). Interestingly, the BBB was not drastically disrupted at either 2 or 6 dpi (Fig. 4B and C), although virus amplification in the brain became evident at 4 dpi. Despite the rapid amplification rates of ZIKV in muscle, viral titers in the brain and SC reached levels higher than in muscle at 6 dpi, which is consistent with the known neurotropism of ZIKV. The detection of the ZIKV negative-strand RNA in the brain, DRG, and SC confirms the active viral replication in the nervous system (Fig. 4D). It is interesting to note that ZIKV replication in the muscle and brain remains constant, maintaining a 2-log difference between these tissues from 6 to 16 dpi (Fig. 4E).

### ZIKV replication in muscle promotes inflammatory infiltration and may compromise regenerative myogenesis.

**FIG 5:**
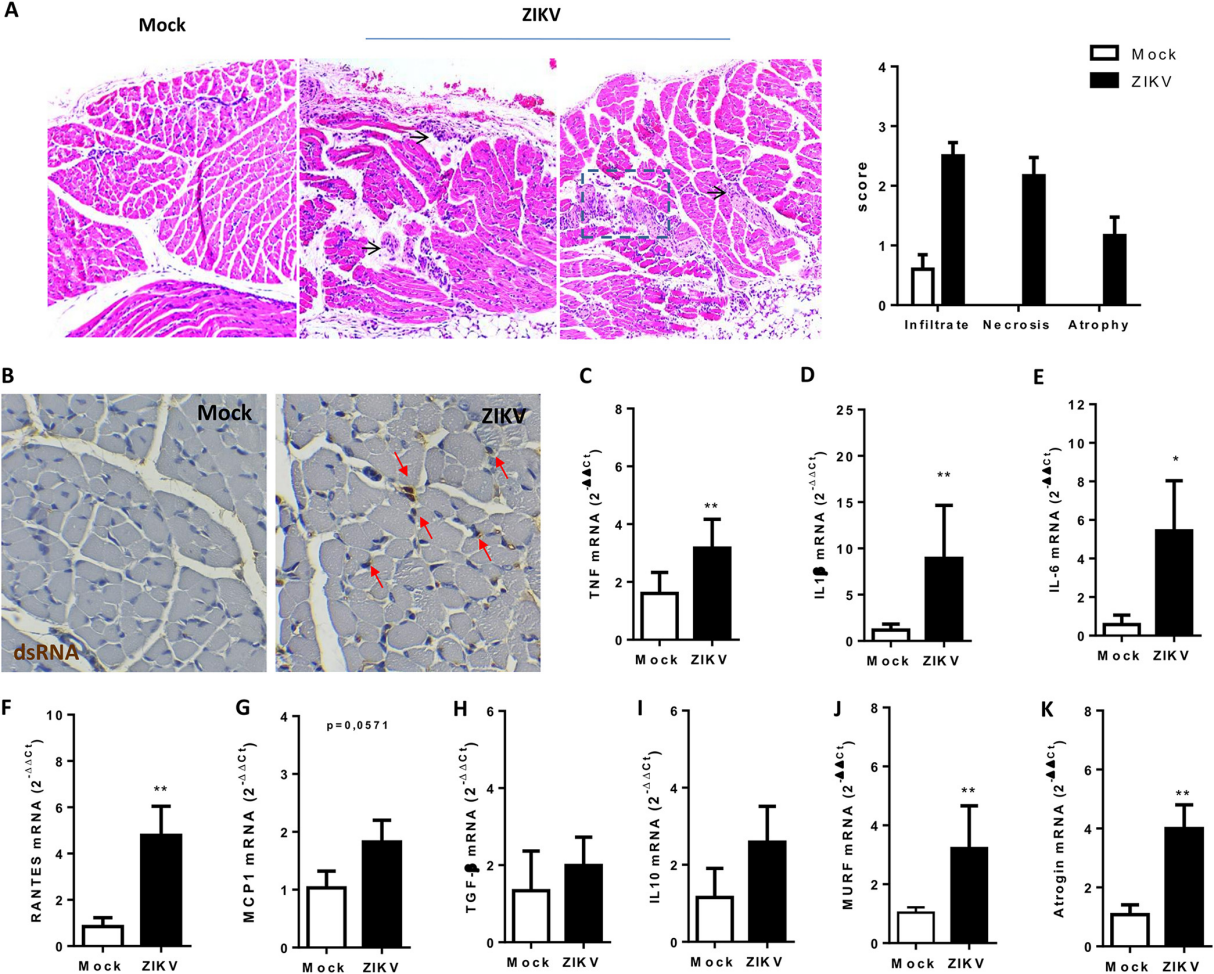
ZIKV induces skeletal muscle inflammatory lesions and atrophy in neonate infection. Three-day-old WT SV129 mice were ZIKV infected (10^6^ PFU), and skeletal muscle tissues from the hind legs were collected at 6 dpi. (A) Muscle samples were embedded in paraffin after dehydration, and 5-μm tissue sections were prepared, stained with H&E, and scored. Black arrows indicate areas of inflammation; dashed lines indicate areas of lesion. (B) The ZIKV replication intermediary double-stranded RNA (dsRNA) was stained in muscle sections at 6 dpi using anti-dsRNA J2 antibody in mock- and ZIKV-infected groups. Slides were imaged on a Sight DS-5M-L1 digital camera (Nikon, Melville, NY) connected to an Eclipse 50i light microscope (Nikon). Red arrows indicate positive staining areas. (C to K) Skeletal muscle from hind legs of neonates was bilaterally collected, and the levels of TNF-α, IL-1β, IL-6, RANTES, MCP-1, TGF-β, IL-10, MURF, and Atrogin expression were determined relative to the mock-infected group by qPCR using β-actin expression as an endogenous control. Values are plotted as means ± the SEM. Statistical analysis was performed using a two-sided Mann-Whitney test (*, *P* < 0.05; **, *P* < 0.01).

Histological analysis of the skeletal muscle of ZIKV-infected neonatal mice showed several areas of inflammatory infiltrates, necrosis, and fiber atrophy (Fig. 5A), as observed in the maternal transmission model. We also detected dsRNA in the muscle tissue of infected mice, which is consistent with ZIKV amplification in the skeletal muscle (Fig. 5B). dsRNA staining could be observed in cells surrounding mature fibers, as indicated by red arrows. In agreement with the findings for cellular infiltrate, we found increased mRNA expression of the proinflammatory cytokines tumor necrosis factor (TNF), interleukin-1β (IL-1β), and IL-6 and also of the chemokines RANTES and MCP-1 in muscle tissues of ZIKV-infected animals (Fig. 5D to G). In contrast, no change in the mRNA levels of anti-inflammatory cytokines transforming growth factor β and IL-10 were seen in skeletal muscle tissue following ZIKV infection compared to mock-treated animals (Fig. 5H and I). RING Finger 1 (MuRF1) and Atrogin-1 are the main ubiquitin ligases involved in the control of protein degradation in muscle, leading to muscle atrophy (17). We found increased expression of both MuRF1 and Atrogin-1 in the muscles of ZIKV-infected mice compared to control mice (Fig. 5J and K), suggesting that ZIKV replication in the skeletal muscle tissue promotes inflammation-induced muscular atrophy.

**FIG 6:**
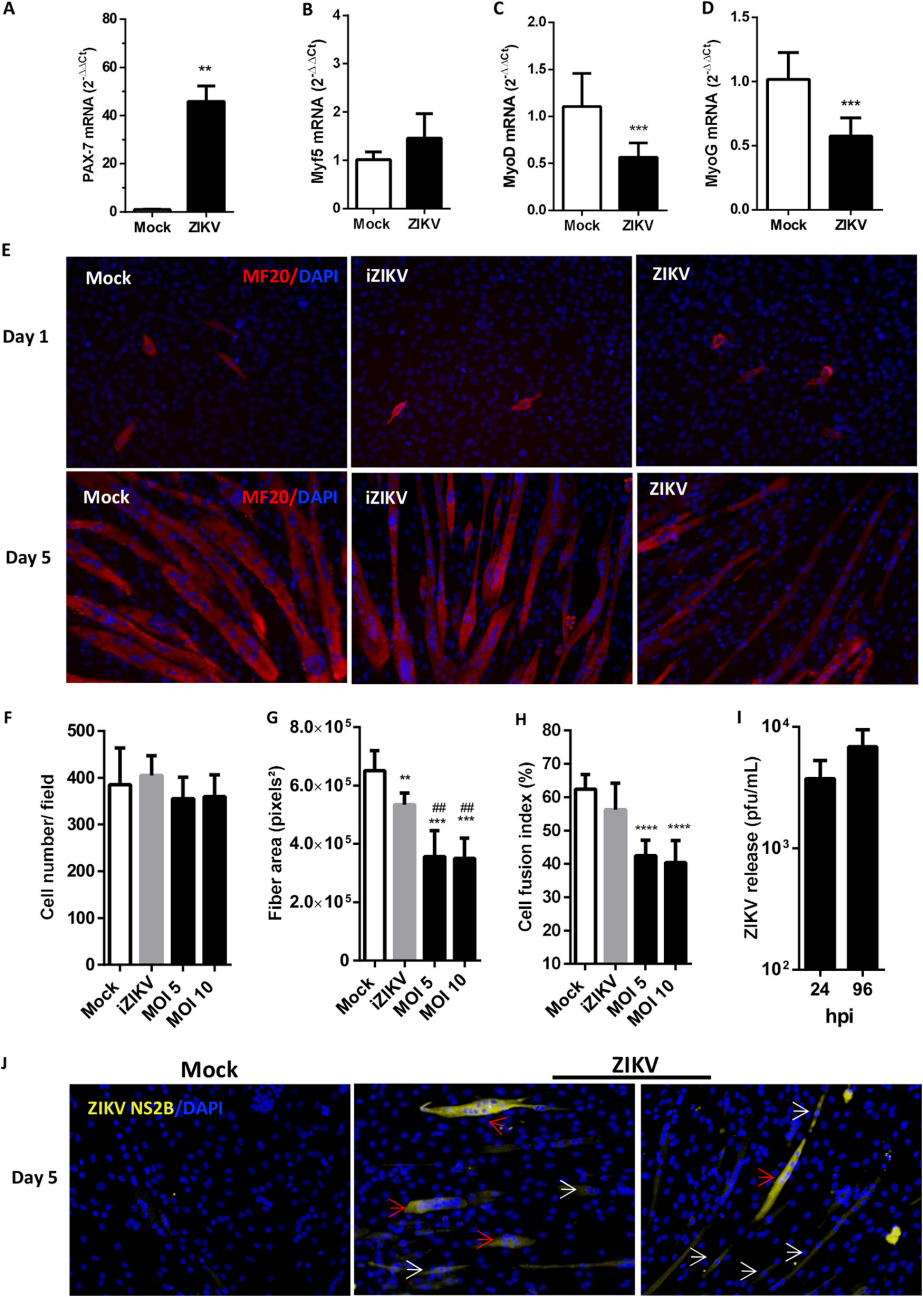
ZIKV impairs activation of myogenic regulatory factors after injury and promoted a replication-dependent reduction in myogenesis in mouse cells. (A to D) Three-day-old WT SV129 mice were ZIKV infected (10^6^ PFU), and skeletal muscle tissues from hind legs were bilaterally collected at 6 dpi. The levels of PAX-7, Myf5, MyoD, and MyoG expression were determined relative to the mock-infected group by qPCR using β-actin expression as an endogenous control. (E) Myoblast C2C12 cells were subjected to differentiation into myotubes. At day 1 of differentiation, the cells were mock treated, inoculated with UV light-inactivated ZIKV (iZIKV), or infected with ZIKV and then cultured until day 5 of differentiation. The cells were fixed at days 1 and 5 of differentiation, and formed fibers were detected by immunofluorescence using MF20 (red); the cell nucleus was stained with DAPI (blue). (F to H) Fluorescence images were quantified using at least 10 fields of three independent experiments with ImageJ software to obtain the cell number per field (F), the fiber area (G), and the fusion index (%) (H) of mock-infected cells (white bars) and ZIKV-infected cells (black bars). Nuclei labeled positive were counted in each field and used to normalize the values per field. (I) ZIKV released in the culture supernatant at different times postinfection was quantified by using a plaque assay (*n* = 5, each point). (J) An immunofluorescence assay was performed to detect NS2B ZIKV protein-positive cells at 5 days of differentiation. Arrows indicate mononucleated (white) and multinucleated (red) positive cells. Values are plotted as means ± the SEM. Statistical analysis was performed using the Mann-Whitney test (A) and one-way ANOVA, followed by Tukey’s multiple-comparison test (F to I) (**, *P* < 0.01; ***, *P* < 0.001 [compared to mock infection]; ##, *P* < 0.01 [compared to iZIKV]).

We also investigated the expression of the sequential myogenic regulatory factors (MRFs) that are essential for myogenesis induction during muscle development and repair. ZIKV infection promoted a significant increase in the mRNA expression of PAX-7 (Fig. 6A), which is responsible for preserving undifferentiated muscle satellite cells but is also an early myogenic activation factor after injury (18). However, ZIKV infection did not interfere with Myf5 mRNA levels (Fig. 6B) and reduced the expression of MyoD (Fig. 6C), two early myogenic commitment factors. Levels of the differentiation inductor factor MyoG were also reduced following ZIKV infection (Fig. 6D). These findings indicate that even though ZIKV activates satellite cells, the skeletal muscle development and formation of new fibers may be compromised in mice. These data were consistent with the reduction in myogenesis observed in ZIKV-infected human myoblast cells under differentiation (Fig. 2).

In order to investigate whether active ZIKV replication is determinant for the reduction of muscle fiber formation, we exposed myoblast C2C12 cells to either infectious or UV-inactivated ZIKV (iZIKV) during differentiation. We observed a massive reduction in the MF20 staining 5 days after differentiation in cells infected with ZIKV at multiplicities of infection (MOIs) of 5 and 10 (Fig. 6E). A reduced MF20 staining was also observed in iZIKV-treated cells compared to mock. This reduction on MF20 staining was not associated with a reduction in cell number after ZIKV or iZIKV treatment (Fig. 6F). Interestingly, iZIKV treatment of C2C12 cells led to a significant reduction in fiber area (Fig. 6G) but did not affect cell fusion index (Fig. 6H), both of which were reduced in cells treated with infectious ZIKV. These results indicate that although the myosin mass gain could be affected by the presence of iZIKV, the inhibition of myoblast fusion is dependent on actively replicating virus. In agreement, ZIKV replicates in C2C12 myoblasts under differentiation, and high viral titers are maintained for at least 96 hpi (Fig. 6I). In addition, ZIKV NS2B immunostaining was also found in C2C12 cells at 5 days of differentiation (about 9.15% of cells [data not shown]). The staining was found not only in some mononucleated cells (white arrows) but also in multinuclear cells (red arrows) (Fig. 6J). Taken together, these results indicate that ZIKV replication in skeletal muscle promotes a proinflammatory process that may interfere with the progression of reparative myogenesis by reducing differentiation commitment and fusion of precursor muscle cells.

## DISCUSSION

ZIKV neurotropism has been extensively characterized due to the potential risk of microcephaly and other birth defects in newborns after vertical transmission (19). However, the contribution of ZIKV replication in peripheral tissues to pathogenesis is still unexplored.

Muscular pain is a recurrent symptom associated with ZIKV infection, affecting up to 65% of patients in some outbreaks (20, 21). Skeletal muscle is the most abundant tissue in the human body, comprised by mature fibers, myogenic precursor cells, and connective tissue (22). Muscle cells have been described as a target for replication of some arboviruses, such as dengue virus (DENV) and chikungunya virus, playing a significant role in their pathogenesis (23, 24). Here, we demonstrate that ZIKV replicates and damages human primary undifferentiated muscle cells *in vitro*, as well as the muscle tissue of fetuses and newborn mice after maternal or neonatal viral transmission. Our data indicate that muscle tissue is a target for ZIKV replication and a site of injury. The ability of ZIKV to infect and damage the skeletal muscle is consistent with the high frequency of myalgia in patients, including not only the self-limited cases but also those that develop neurological complications (20, 21, 25).

Although ZIKV tropism for peripheral tissues has been underexplored, clinical studies have shown the viral persistence in several body fluids (16). Initial reports evaluating cell permissiveness have demonstrated that ZIKV replicates at placental trophoblasts, endothelial cells, human skin fibroblasts, and neonatal keratinocytes (16, 26, 27). Consistent with skin lesions presented in many ZIKV-infected patients (28), ZIKV also replicates and induces morphological alterations in human skin explants (26). Analysis of the tissues of SVA129 mice infected at 12.5 days of pregnancy showed a broad distribution of ZIKV after delivery. However, the absence of a preferential site for ZIKV replication could be related to the lack of IFN signaling in SVA129 mice. When ZIKV is inoculated in humans by mosquitoes, skin cells (keratinocytes and dendritic cells) are the first targets of the virus, followed by virus dissemination. Mosquito bite or subcutaneous inoculation in nonhuman primates showed that ZIKV migrates to different organs and tissues, with the highest loads at the lymph nodes (29). In contrast to DENV infection, *in vitro* and *in vivo* studies indicate that the liver does not sustain high rates of ZIKV amplification in animal models (29), even though some hepatocyte lineages were shown to be susceptible (30). Accordingly, we did not find ZIKV amplification in the liver during neonatal infection in wild-type mice, whereas replication in skeletal muscle could be observed as early as 1 dpi. Thus, our data suggest that the skeletal muscle is an important site for ZIKV amplification outside the CNS, especially compared to the liver and spleen.

In agreement with our findings, it was recently demonstrated that undifferentiated myoblast cells were more susceptible to ZIKV infection than are differentiated fibers (31). In neural tissues, ZIKV preferentially replicates in neural progenitor cells disrupting neurogenesis (32–34). These findings reveal that the preference for undifferentiated cells is a common aspect of ZIKV tropism for both muscle and neural tissues. Fetal and neonatal muscle tissues are formed by fibers under maturation and muscle precursor cells, which differ according to their proliferative and fusogenic activities (35, 36). We also demonstrated that ZIKV infection during myoblast differentiation reduces the number and area of the newly formed fibers, indicating that ZIKV replication may impair skeletal muscle myogenesis causing muscle damage during fetal and neonatal muscle development.

In addition to being an important site of ZIKV replication outside the CNS, the skeletal muscle showed a rapid and sustained kinetic of ZIKV amplification even before replication in the neural tissues. Furthermore, the inability of ZIKV to disturb the BBB permeability during early life infection reinforces the possibility that the virus accesses the CNS by peripheral neurons. This is also supported by the detection of ZIKV negative-strand RNA in the DRG of neonates (6 dpi) and corroborates previous studies that demonstrated ZIKV replication in explants and neurons from DRG obtained from ZIKV-infected mice (12, 37). In addition, it was previously demonstrated that ZIKV reaches the CNS by transcytosis, interfering in its permeability only at late stages of infection (11). However, this cannot not exclude that ZIKV uses peripheral neural route to access the CNS, as already described for others neurotropic viruses (13–15). Our data also suggest that this initial stage of viral replication in the skeletal muscle may be a common pathway used by ZIKV and other viruses to reach the neural structure.

Mature muscular fibers are not self-renewable, but a high number of precursor cells are preserved in muscle tissue and are responsible for muscle repair after injury (38). Thus, these precursor cells could be responsible for peripheral ZIKV amplification, even in adult individuals. In our model of neonatal infection, ZIKV titers in muscle were maintained at high levels at least until 16 days postinfection, raising the possibility that this tissue is a site of viral persistence and sustained inflammation. ZIKV dsRNA was mostly detected in areas of inflammation and muscular atrophy, mainly after maternal transmission. However, ZIKV replication was also observed in preserved sites in the neonatal model. ZIKV dsRNA was observed in the cells surrounding mature fibers, which could be either satellite cells or proliferative myoblasts induced by injury. ZIKV infection promoted muscle lesions, with infiltration of inflammatory cells, along with an upregulation of proinflammatory cytokines (TNF, IL-1β, and IL-6) and chemokines (RANTES and MCP-1) involved in monocyte/macrophage recruitment (39–41). Interestingly, the expression of genes involved in muscle protein degradation (atrophy genes) was also increased during ZIKV infection in neonate mice. The acceleration of muscle mass loss could be triggered by several proinflammatory cytokines and other inflammatory mediators, such as TNF and IL-1β (42). In addition, the expression of anti-inflammatory cytokines that are associated with muscle tissue repair, such as TGF and IL-10 (43, 44), was not affected by ZIKV infection.

It is well established that myeloid cell infiltration after muscle injury triggers the activation of an inflammatory process that is not only responsible for muscle degeneration but also for the activation of satellite cells for the formation new fibers promoting tissue repair (45). Activation of quiescent satellite cells results in a signaling cascade that induces MRFs, promoting early proliferation, following commitment to a myogenic program of differentiation and fusion (22, 45). Thus, it would be expected that ZIKV-induced muscle damage and inflammation could acts as a signal that triggers a muscle repair cascade. However, *in vitro* evidence of the inhibition of myogenesis by ZIKV led us to investigate PAX-7, MyF5, MyoD, and MyoG expression levels in infected muscle. PAX-7 regulates the survival and proliferation of satellite cells but is also required for the early activation of MRFs such as MyF5 and MyoD (18). The induction of PAX-7 expression by ZIKV infection shows that it promotes the activation of satellite cells. However, the absence of the induction of MyF5 and MyoD gene expression after PAX-7 stimulation could indicate that ZIKV blocks the progression of reparative myogenesis. In agreement with this, the expression of the differentiation regulatory factor MyoG was also inhibited by ZIKV infection despite the occurrence of muscle damage in mice. PAX-7-dependent myogenic commitment is induced by the recruitment of a histone methyltransferase complex, promoting MRF expression (46), that could be directly or indirectly affected by ZIKV replication as a consequence of cellular antiviral response, such as IFN stimulation of IFN secretion and signaling. Accordingly, treatment of murine C2C12 myoblast cells with type I IFN was able to reduce myotube formation (47). Interestingly, using C2C12 cells undergoing differentiation, we showed that incubation with iZIKV was able to reduce the caliber of newly formed fibers but that cell fusion inhibition was observed only after exposure to infectious ZIKV. We found that ZIKV replication is maintained in C2C12 cultures after 96 h of infection, and ZIKV NS2B protein could be detected in both undifferentiated and multinucleated cells. It is possible that multinucleated NS2B-positive cells could result from cells infected early or from undifferentiated myoblasts. However, only about 9% of cultured infected C2C12 cells are positive for ZIKV NS2B at 5 days of differentiation, indicating that the inhibition of fusion could also occur in uninfected bystander myoblast cells. These evidences of ZIKV persistence, inducing muscle inflammation and the disfunction in repair machinery, raise the possibility that exposure to ZIKV early in life would affect development even outside the neuronal tissue. Further studies should be performed in order to evaluate whether ZIKV infection compromises long-term muscle development and function.

In conclusion, our findings suggest that persistent ZIKV replication in the skeletal muscle, as well as infection-induced inflammation in this tissue, could be an important step in ZIKV pathogenesis. We highlight the importance here of investigating the molecular aspects associated with ZIKV-muscle interactions during the initial steps of the infection, its consequences for virus dissemination in the body, and its relationship to CNS damage in ZIKV-infected patients. Of note, the control of viral replication in the muscle could be an important aspect for the development of therapeutic strategies to prevent neurological complications after ZIKV infection. Taken together, our data contribute to a broader understanding of ZIKV pathogenesis and open new aspects for future investigations of ZIKV-induced injuries.

## MATERIALS AND METHODS

### Virus propagation.

Zika virus (BRPE243/2015, KX197192) was propagated in the C6/36 cell lineage cultured in Leibovitz L-15 medium (Invitrogen) supplemented with 10% fetal bovine serum (FBS) at 28°C. C6/36 cells were infected at an MOI of 0.01; after 7 days, the culture medium was collected and stored at −80°C. The same procedure was performed using C6/36 uninfected cells to allow the production of the “mock” stock. The titer of viral stock was determined by plaque assay in Vero cells cultured in high-glucose Dulbecco modified Eagle medium (DMEM; Invitrogen) after 10-fold serial dilutions. Inactivated ZIKV (iZIKV) was obtained by exposing the stock solution to UV light in open dishes for 40 min. Viral inactivation was confirmed by plaque assay in Vero cells.

### Muscle cell culture, differentiation, and infection.

HSMM cells (human skeletal muscle myoblasts) are precursor skeletal muscle cells isolated from the arms or legs of adult healthy donors. Stocks were obtained from Lonza cell facility (catalog number CC-2580). The C2C12 cell lineage, a murine myoblast cell lineage, was kindly donated by Flávia Bloise (IBCCF-UFRJ, Brazil). HSMM cells were cultured according to Lonza specifications (using SkGM medium), while C2C12 cell were cultured in DMEM supplemented with 10% FBS and then maintained at 37°C and 5% CO_2_. To induce differentiation of myoblasts into myotubes, cells were plated at high density, and SkGM or DMEM were supplemented with 2% horse serum (GIBCO/Life Technologies). The culture medium was replaced every 2 days until fiber formation (ca. 5 to 6 days).

HSMM myoblasts and myotubes were infected with ZIKV at an MOI of 5 in SkGM without serum for 1 h. After infection, HSMM cells were cultured in SkGM supplemented with 5% FBS. The culture medium was collected at different times to determine the amount of ZIKV released by plaque assay. Myoblast and myotube viabilities were determined using MTT [3-(4,5-dimethylthiazol-2-yl)-2,5-diphenyltetrazolium bromide; Life Technologies] metabolization.

For infection during differentiation, HSMM or C2C12 cells were cultured as described above. After 1 day of differentiation stimuli (day 1), HSMM or C2C12 cells were mock treated or treated with ZIKV or iZIKV and then cultured in differentiation medium until they completed 5 days of stimulation (day 5). The culture medium was collected at different time points to quantify the infectious ZIKV in the medium by a plaque assay. Cells were fixed at days 1 and 5 and used for immunostaining for different targets.

### Fluorescence microscopy.

Cells seeded in 24-well plates were fixed with 4% formaldehyde in phosphate-buffered salt solution (PBS; pH 7.4) at the desired times postinfection. Staining for viral proteins was performed with conditioned 4G2 hybridome (anti-flavivirus mouse antibody) media at a 1:10 dilution or ZIKV NS2B protein antibody (rabbit polyclonal IgG; Genetex, catalog no. GTX133308) at a 1:100 dilution. Myofibers were stained using a polyclonal antibody against myosin heavy chain (MF20) at a 1:50 dilution. They were then stained with goat anti-mouse Alexa Fluor 488 (Invitrogen) at a 1:500 dilution. Cells in each field were visualized with the nuclear stain DAPI (GIBCO/Life Technologies) at a 1:10,000 dilution. Images were acquired using an inverted fluorescence microscope (IX81; Olympus) at a magnification of ×20. Images of 4G2-positive cells for at least 10 fields were used to determine the percentages of infected HSMM cells. The cell fusion index in cultured mock-treated or ZIKV-infected myotubes was determined by quantification of MF20-positive cells as the percentage of the total number of nuclei (DAPI), and the fiber area was obtained by measuring the MF20 fluorescence normalized by the cell number per field. Image quantification was performed for at least 10 fields using ImageJ software (version 1.51 j.8).

### Mouse infection and tissue samples.

All experimental procedures using animals were performed in accordance with the protocol and standards established by the National Council for Control of Animal Experimentation (CONCEA, Brazil) and approved by the Institutional Animal Care and Use Committee (CEUA) of the Federal University of Rio de Janeiro (protocol 014/16; CEUA-UFRJ, Rio de Janeiro, Brazil).

For the mouse model of ZIKV maternal transmission, we used 8-week-old type I IFNAR^−/−^ pregnant mice at different gestational stages (12.5, 14.5, and 18.5 days of gestation). Pregnant mice were subcutaneously inoculated in the left footpad with the mock treatment or 10^5^ PFU of ZIKV in a final volume of 50 μl and then monitored until delivery. At postnatal day 0 (P0), the numbers of live-born pups were counted, and vital signs were observed. Pups were killed by decapitation, and their brains and skeletal muscle tissues from left and right hind legs were collected and either fixed in 4% formaldehyde for histological analysis or stored at −80°C for viral quantification analysis.

For the neonatal infection mouse model, 3-day-old WT SV129 mice were subcutaneously inoculated with either mock treatment or 10^6^ PFU of ZIKV in the dorsum in a final volume of 50 μl. Each experimental group was subjected to the same treatment to avoid cross-contamination and housed with the uninfected mother in polypropylene cages maintained at 25°C with controlled humidity, under a 12-h light/dark cycle with free access to chow and water. Mice were monitored daily, and the body weight was measured every 2 days. Tissue samples of mock- and ZIKV-injected mice were collected at different time points and stored at −80°C until they were processed for qPCR analysis or fixed in 4% formaldehyde for histological analysis.

### Endothelial barrier integrity analysis.

Neonate SV129 mice were subcutaneously inoculated with 50 μl of 0.1% Evans Blue solution (VETEC, Brazil) before (3 days old, moment of infection) and at 2 and 6 days after the injection of mock treatment or ZIKV. After 1 h of dye injection, mice were anesthetized and perfused with PBS, and their brains and peripheral tissues (liver, spleen, and muscle) were collected to observe Evans Blue staining. For quantification of Evans Blue staining, at 6 dpi, the brains were removed, weighed, and then stored in 1 ml of formamide at room temperature for 3 days. The optical density (OD) of the dye resuspended in formamide was measured using a spectrophotometer at 620/680 nm (SpectraMax i3). The amount of Evans Blue dye that crossed BBB and reached the brain was determined by correlation with the OD from a standard curve and plotted as μg of Evans Blue dye/mg of tissue.

### Histology and immunohistochemistry.

Skeletal muscle samples from mouse hind legs were collected bilaterally at birth or 6 dpi, fixed with 4% paraformaldehyde, and embedded in paraffin after dehydration. Paraffin-embedded tissue sections of 3 to 5 μm were prepared and stained with hematoxylin and eosin (H&E) or used for immunohistochemistry. H&E images were obtained by using optical microscopy at a magnification of 10× (Olympus BX40), and images were acquired using Leica Application Suite 3.8 software. For scoring, mock-infected or ZIKV-infected skeletal muscle tissues stained with H&E from at least five different animals were classified for the presence of necrosis, cellular infiltrate, and fiber atrophy by a researcher blind to the experimental condition, according to the following criteria: 0, absence; 1, some indicative; 2, moderate; 3, intense; and 4, very intense.

For immunohistochemistry of ZIKV dsRNA, sections of skeletal muscle were stained as previously described (8) with primary mouse anti-dsRNA antibodies (SCICONS J2) at a 1:200 dilution. Stained tissue sections were analyzed under identical conditions to allow comparison between immunoreactivity ODs. Slides were imaged on a Sight DS-5M-L1 digital camera (Nikon, Melville, NY) connected to an Eclipse 50i light microscope (Nikon).

### Quantification of ZIKV RNA and gene expression by qPCR.

Tissues were homogenized in DMEM (0.2 mg tissue/μl), and 200-μl portions of the homogenate were used for RNA extraction with TRIzol (Invitrogen) according to the manufacturer’s instructions. The purity and integrity of the RNA were determined by using 260/280- and 260/230-nm absorbance ratios. A portion (1 μg) of isolated RNA was subjected to DNase I treatment (Ambion; Thermo Fisher Scientific, Inc.) and then reverse transcribed using a high-capacity cDNA reverse transcription kit (Thermo Fisher Scientific, Inc.).

To quantify the ZIKV, the following RNA primers for ZIKV described by Lanciotti et al. (48) were used: forward, 5′-CCGCTGCCCAACACAAG-3′; reverse, 5′-CCACTAACGTTCTTTTGCAGACAT-3′; and probe, 5′-/56-FAM/AGCCTACCT/ZEN/TGACAAGCAATCAGACACTCAA/3IABkFQ/-3′ (Integrated DNA Technologies). Analyses were carried out using an Applied Biosystems 7500 RT-PCR system with TaqMan Mix (Thermo Fisher Scientific, Inc.) according to the manufacturer’s instructions. Cycle threshold (*C_T_*) values were used to calculate the equivalent (Eq.) of PFU/μg of total RNA after conversion using a standard-curve with serial 10-fold dilutions of ZIKV stock.

To detect ZIKV negative-strand RNA, total RNA was extracted as described above, and cDNA was synthesized using 2 pmol of ZIKV 835 forward primer instead of random primers. Real-time quantitative PCR analysis was performed as described above.

Quantification of gene expression in the skeletal muscle was performed using a Power SYBR kit (Applied Biosystems, Foster City, CA). Actin was used as an endogenous control. The primer sequences were as follows: IL-6 (FW, 5′-TTCTTGGGACTGATGCTGGTG-3′; REV, 5-CAGAATTGCCATTGCACACTC-3′), MCP-1 (FW, 5′-GTCCCCAGCTCAAGGAGTAT-3′; REV, 5′-CCTACTTCTTCTCTGGGTTG-3′), RANTES (FW, 5′-GTGCCCACGTCAAGGAGTAT-3′; REV, 5′-CCTACTTCTTCTCTGGGTTG-3′), TNF (FW, 5′-CCTCACACTCAGATCATCTTCTCA-3′; REV, 5′-TGGTTGTCTTTGAGATCCATGC-3′), IL-1β (FW, 5′-GTAATGAAAGACGGCACACC-3′; REV, 5′-ATTAGAAACAGTCCAGCCCA-3′), TGF-beta (FW, 5′-GAC CGC AAC AAC GCC ATC TA-3′; REV, 5′-AGC CCT GTA TTC CGC CTC CTT-3′), IL-10 (FW, 5′-TAA GGG TTA CTT GGG TTG CCA AG-3′; REV, 5′-CAA ATG CTC CTT ATT TCT GGG C-3′), MURF1 (FW, 5′-GAGAACCTGGAGAAGCAGCTCAT-3′; REV, 5′-CCGCGGTTGGTCCAGTAG-3′), ATROGIN-1 (FW, 5′-AGAAAAGCGGCACCTTCGT-3′; REV, 5′-CTTGGCTGCAACATCGTAGTT-3′), Pax-7 (FW, 5′-GAA TCA GAA CCC GAC CTC CC -3′; REV, 5′-CGC CGG TTA CTG AAC CAG A-3′), Myf5 (FW, 5′-TGA GGG AAC AGG TGG AGA AC-3′; REV, 5′-AGC TGG ACA CGG AGC TTT TA-3′), MyoD (FW, 5′-TTC TTC ACC ACT CCT CTG ACA-3; REV, 5′-GCC GTG AGA GTC TTA ACTT-3′), MyoG (FW, 5′-ATC CAG TAC ATT GAC CGC CT-3; REV, 5′-GCA AAT GAT CTC CTG GGT TG-3′), and Actin (FW, 5′-TGTGACGTTGACATCCGTAAA-3′; REV, 5′-GTACTTGCGCTCAGGAGGAG-3′).

### Statistical analyses.

Statistical analyses were performed using Prism version 7.00 for Windows (GraphPad Software, La Jolla, CA). The tests used are indicated in the corresponding figure legends. Briefly, multiple groups were analyzed using one-way or two-way analysis of variance (ANOVA), followed by multiple-comparison analyses. To compare means, we used a nonparametric Mann-Whitney test.
